# Effect of reflection and refraction on NEXAFS spectra measured in TEY mode

**DOI:** 10.1107/S1600577517016253

**Published:** 2018-01-01

**Authors:** Elena Filatova, Andrey Sokolov

**Affiliations:** a Institute of Physics, St Petersburg State University, Ulyanovskaya Strasse 3, St Petersburg 198504, Russian Federation; b Helmholtz-Zentrum Berlin für Materialien und Energie GmbH, Albert Einstein Strasse 15, Berlin 12489, Germany

**Keywords:** NEXAFS, total electron yield method, X-ray reflection spectroscopy

## Abstract

The evolution of near-edge X-ray absorption fine structure in the vicinity of the *K*-absorption edge of oxygen for HfO_2_ over a wide range of incidence angles is analyzed by simultaneous implementation of the total-electron-yield (TEY) method and X-ray reflection spectroscopy. It is established that the effect of refraction on the TEY spectrum is greater than that of reflection and extends into the angular region up to angles 2θ_c_. Within angles that are less than the critical angle, both the reflection and refraction strongly distort the shape of the TEY spectrum. Limitations of the technique for the calculation of optical constants from the reflection spectra using the Kramers–Kronig relation in the limited energy region in the vicinity of thresholds are discussed in detail.

## Abstract 2

A study of the effect of reflection and refraction processes on the evolution of the total-electron-yield spectrum over a wide range of incidence angles including grazing angles is reported.

## 1. Introduction   

Progress in the development of new X-ray sources such as synchrotrons of the third and, in the nearest future, fourth generation and free-electron X-ray lasers in many respects determines now the trends of development of atomic physics, condensed matter physics, chemical physics, materials science and physics interactions of high-frequency electromagnetic radiation with matter. Creating a super-high-resolution monochromator at soft X-ray wavelengths and electronic analyzers can significantly increase the informative character of the study of electronic and atomic structure and chemical bonding of polyatomic compounds by spectroscopy core levels (Stöhr, 1992 ▸; de Groot & Kotani, 2008 ▸; Suga & Sekiyama, 2013 ▸), and makes these techniques indispensable tools for investigation in various fields of modern science and technology. Methods of the core-level spectroscopy family (absorption, emission and reflection X-ray spectroscopies, photoelectron and Auger-electron spectroscopies) are now the most widely used methods for studying the electronic and atomic structure of matter, engaging the top modern experimental technologies (Penner-Hahn, 2003 ▸; Stoupin *et al.*, 2016 ▸; McFarland *et al.*, 2014 ▸).

X-ray spectroscopy includes all the spectral methods based on transitions between states of a system, which has core-holes (the hole on the inner shell of the atom) in the initial, final or intermediate state. The undeniable advantage of these methods is their high sensitivity to the local electronic and atomic structure of the object. This makes it equally applicable to the study of structure from isolated atoms and simple molecules to complex biological molecules, clusters, nano­structures, surface and bulk solids (Erbahar *et al.*, 2016 ▸; Aetukuri *et al.*, 2013 ▸; Ye *et al.*, 2015 ▸).

It is well known that the characteristics of chemical bonding in a polyatomic system and, as a consequence, its atomic and electronic structure are alike determined by the characteristics of upper occupied (mainly bonding states) and low-lying unoccupied (usually non-bonding states) electronic states of the system. In particularly, near-edge X-ray absorption fine structure (NEXAFS) (Stöhr, 1992 ▸) arises from excitations into unoccupied molecular orbitals, and is dominated by multiple scattering of a low-energy photoelectron in the valence potential formed by the nearest surroundings that defines the highest sensitivity of NEXAFS to distinguish chemical bonds and nearest surrounding atoms. Thus NEXAFS provides information about local (associated with a hole localization in the core shell) and partial (allowing for certain angular momentum symmetry) electronic density of states of the conduction band. NEXAFS analysis in the ultra-soft X-ray region from 50 to 1000 eV is a technique of great importance for studying the local structure and electronic states of many elements in important functional materials, since in this region the absorption edges of the *K* shell of light elements from Li to C, N and O and the *L* shell of transition metals exist, which are often key elements.

While NEXAFS spectra in the hard X-ray region have most conveniently been obtained in transmission mode, soft NEXAFS spectra have normally been obtained by detecting emitted electrons or photons due to the difficulty of preparing thin enough samples corresponding to their extremely low transmittance in the soft X-ray region. The total electron yield (TEY) method, which does not require special thin sample preparation, is a well established method for measuring NEXAFS spectra. This technique consists of collecting electrons of all energies that emit from the sample surface by measuring the drain current from the irradiated sample. Alternatively, when the electrical decoupling of the sample from the grounded experimental chamber is not possible, one can collect electrons using a channeltron detector placed close to the irradiated area on the sample. Acceptance in this case is limited by detector aperture, but, in general, the measured signal should be similar to that collected in drain current mode. In the present work, TEY spectra were obtained by measurement of drain current from the sample. Usually TEY spectra give a spectral shape similar to that of the absorption spectra. Nevertheless, TEY spectra can exhibit distortions when the electron escape depth is comparable with the X-ray penetration depth. A simple model (Stöhr, 1992 ▸; Shchemelev & Savinov, 1998 ▸) is usually used to describe TEY that averages over the various complicated electron scattering processes and describes them by effective parameters with physical meaning. According to Henke (1972 ▸), in the soft X-ray region at sufficiently large glancing angle (>10°) we can neglect reflection and refraction and express the absorption coefficient μ(θ,ω) in terms of the regular absorption coefficient μ(ω). In the framework of this model the interaction of the X-rays with the bulk sample can be expressed by an absorption coefficient μ(θ,ω),
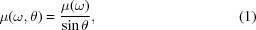
where θ is the X-ray grazing incidence angle on the sample measured from the surface (Stöhr, 1992 ▸; Shchemelev & Savinov, 1998 ▸). It is important that the so-defined absorption coefficient does not include the effects of X-ray refraction at the surface, and in general it can be obtained from the optical constants (Henke *et al.*, 1982 ▸; Veigele, 1973 ▸) derived for free atoms.

The spectral dependence of the absorption coefficient μ(ω) can be obtained by using X-ray reflection spectroscopy. Soft X-ray total external reflection is an integrated process and is defined by both the photoabsorption process and the polarizability of the substance. The primary photon 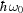 impinges on a surface of the sample and photoionization takes place; locally a photon is absorbed and an electron is excited into unoccupied states. The interaction of the photon 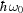 with the sample leads to the polarization of the substance. As a result the system returns to the undisturbed state when the core-hole is filled and the photon is emitted. The intensity of the ‘reflected’ photons is detected. It is important that the reflection coefficient measured in the soft X-ray region contains information about the optical constants (refraction and absorption coefficients) of the substance. The spectral dependencies of the reflection coefficient (reflection spectra) *R*(ω) exhibit ‘edges’ specific to the elements present in the sample and are very sensitive to the type of absorbing atom, their chemical state and the local coordination environment. Fine structure of reflection spectra arising in the vicinity of absorption edges provides information about local (associated with a hole localization in the core shell) and partial (allowed for certain angular momentum symmetry) electronic density of states of the conduction band. Note that different methods allow optical constants to be extracted from reflectivity measurements (Sève *et al.*, 1998 ▸; Wang *et al.*, 2005 ▸; Al-Marzoug & Hodgson, 2007 ▸; Windt *et al.*, 1988 ▸; Scott, 1998 ▸) but all of them are based on the simulation of optical constants from angular dependencies of the reflectivity. We focus on the method developed by us (Filatova *et al.*, 1999 ▸; Filatova *et al.*, 2010 ▸) and based on the calculation of the absorption and refraction spectral dependencies from measured reflection spectra. Only implementation of the Kramers–Kronig transform allows the spectral dependencies of the optical constants to be derived from the measured reflection spectra. At the same time, the Kramers–Kronig transform needs a large range of integration and as a consequence extrapolations outside the experimental energy range need to be applied. Despite the careful mathematical analysis of the complexity of such an approach (Toll, 1956 ▸; Stern, 1963 ▸; Young, 1977 ▸; Plaskett & Schatz, 1963 ▸; Vinogradov *et al.*, 1988 ▸; Sheik-Bahae, 2005 ▸; Lucarini *et al.*, 2005 ▸; King, 2009 ▸), up to now there has been no experimental confirmation of the identity of the shape of the near-edge structure of measured absorption spectra and the absorption spectra calculated from reflection spectra. This work is a first experimental attempt to clarify this problem. Moreover, such an approach allows us to derive the absorption spectra from the reflection spectra over a wide angular region and then to trace experimentally the evolution of the shape of measured and calculated TEY spectra with glancing angle simultaneously with the reflection spectra measured at the same angles. Such analysis makes it possible to find the angular region where absorption spectra do not feel the influence of reflection and refraction.

## 2. Experimental details   

The angular and spectral dependencies of the reflection coefficients and TEY spectra as a drain current from the sample were measured using s-polarized synchrotron radiation in the polarimeter set up on the UE56-2/PGM-2 beamline at the Berlin Synchrotron Radiation facility, BESSY II, of the Helmholtz-Zentrum Berlin (Schäfers *et al.*, 1999 ▸; Sawhney *et al.*, 1997 ▸). A GaAsP diode together with a Keithley 6514 electrometer was used as a detector. We used a detector with a 4 mm-diameter window to monitor the total intensity of the reflected beam. The energy resolution at 510 eV was better than 150 meV (the energy resolving power was *E*/Δ*E* = 3400). The relative magnitude of the statistical fluctuations of the reflection coefficient was of the order of 0.02% in the case of reflection spectra measured at 2° and 3°. The drain current from the sample was measured using a Keithley 6514 electrometer. An iron absorption filter was used to reduce high diffraction orders in the incident beam.

The HfO_2_ film of 5 nm thickness, synthesized by the metallo-organic chemical vapour deposition method, onto a p-type Si(100) surface was investigated. Tetra­kis­diethyl­amino­hafnium {Hf[(CH_3_)_2_N]_4_} and oxygen (O_2_) were used as precursors. The temperature of the substrate was 485°C.

## 3. Calculation details   

### 3.1. TEY method   

In the simple model framework (Shchemelev & Savinov, 1998 ▸; Stöhr, 1992 ▸) the TEY in current mode χ_c_ is defined as the total number of electrons emitted from the sample (*n*_B_) per incident photon *N*_0_ and can be expressed as
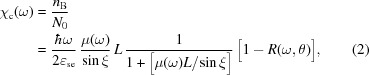
where μ(ω) is the linear absorption coefficient, *R*(ω,θ) is the reflection coefficient, θ is the glancing incidence angle, ξ is the glancing refraction angle, ∊_se_ is the average energy needed to create a secondary electron that is able to escape in a vacuum from the photocathode, 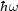 is the photon energy, 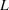 = 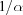 is the effective electron drift length, and α is the attenuation coefficient of the outgoing electrons (decrease in the number of electrons moving in the selected direction due to scattering) and is, to first order, independent of the photon energy. As follows from formula (2), if 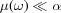 or 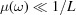 then
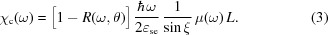
This means that, in the range of large grazing incidence angles when 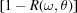 ≃ 1, one can assume that 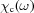 ≃ 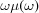, which is the basis of ‘yield spectroscopy’. This was first pointed out by Lukirskii (Lukirskii & Brytov, 1964 ▸; Gudat & Kunz, 1972 ▸). Note that *L* can be estimated from experiment with a simple formula derived from (2) by using two values of the electron yield measured at different incident angles θ_1_ and θ_2_, which are large enough to neglect by changing the reflection coefficient and the difference between the refraction and incident angles,
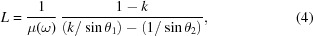
where 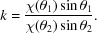
As follows from formulas (2) and (3), TEY is strongly enhanced at grazing incidence angles and it is no longer proportional to the X-ray absorption coefficient. This was firstly observed experimentally by Gudat (1974 ▸) and Martens *et al.* (1978 ▸). The physical reason for this effect is that all X-rays are absorbed in a surface layer of thickness 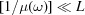 and the electron signal saturates.

### 3.2. X-ray reflection spectroscopy   

The Fresnel reflection coefficient *r* for the specular reflected s-polarized radiation is given by 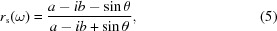
where
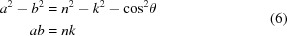
and θ is the glancing angle. In the experiment, the reflection coefficient 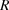 = 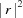 for intensities is measured. The amplitude of the complex coefficient of reflection *r* can be expressed in terms of its modulus 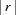 = 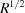 and its phase shift ψ appearing after reflection of the wave at the surface of the medium. Then one can deduce
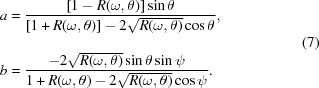
The reflection coefficient *R* and phase shift ψ are linked by the Kramers–Kronig dispersion relation,
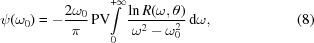
where PV denotes the principal value of the integral. It is known (Toll, 1956 ▸; Stern, 1963 ▸; Young, 1977 ▸; Plaskett & Schatz, 1963 ▸; Vinogradov *et al.*, 1988 ▸; Sheik-Bahae, 2005 ▸; Lucarini *et al.*, 2005 ▸; Nash *et al.*, 1995 ▸; Smith, 1977 ▸; Lee & Sindoni, 1997 ▸; André *et al.*, 2010 ▸) that generally an integral in the form (8) ambiguously defines the dependence between the imaginary and real parts of a function, *i.e.* additional arbitrary constants (zero-frequency phase term, Blashke factors) along with their own dispersive integral are possible. As an example, the Blashke factors correspond to the occurrence of singularities in the complex coefficient of reflection *r* along the imaginary frequency axis. As follows from the literature (Toll, 1956 ▸; Stern, 1963 ▸; Young, 1977 ▸; Plaskett & Schatz, 1963 ▸; Vinogradov *et al.*, 1988 ▸; Sheik-Bahae, 2005 ▸; Lucarini *et al.*, 2005 ▸; Nash *et al.*, 1995 ▸; Smith, 1977 ▸; Lee & Sindoni, 1997 ▸; André *et al.*, 2010 ▸), the value of additional terms depends strongly on the behavior of the dielectric constant and on the conditions of the measurements such as oblique or normal incidence, type of the polarization of the incident beam, material of the sample and nature of the mirror (massive or layered system). Nevertheless, in the case of an isotropic massive film and s-polarized radiation, the phase shift ψ, appearing after reflection of the wave at the surface of the mirror, changes in the limits 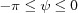. Then, according to analysis of the appearance of additional constants in (8) (Toll, 1956 ▸; Stern, 1963 ▸; Young, 1977 ▸), the phase shift can be uniquely deduced from reflection coefficients *R* using (8). As follows from (8), the integral covers the whole energy range, and the uncertainty in the determination of the phase shift is mainly caused by the limitation of the energy range covered by the reflectivity measurements. It is convenient to rewrite the integral (8) as
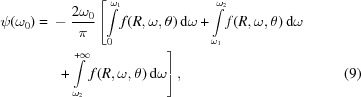
where the energy region (ω_1_, ω_2_) corresponds to the domain of the measurement. As follows from (9), outside the spectral range of the experiment, extrapolations of the reflection spectra have to be used.

Some interesting consequences of the dispersion relation (8) can be established using integration by parts and rewriting (8) in the form
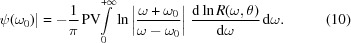
Analysis of the integrand in (10) indicates that function 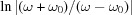 has a strong peak at ω = ω_0_ and spectral regions where the function *R*(ω) changes rapidly that leads to a high value of the derivative 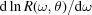. This means that the main contribution to the value of the phase shift ψ(ω_0_) gives spectral regions adjacent to the point ω = ω_0_. With reference to ultrasoft X-ray spectra this means that the extrapolation to the hard X-ray spectral region (ω > ω_2_) where the reflection coefficient typically decreases sharply, and to the UV spectral region (ω < ω_1_) where the oscillations of *R*(ω) related to the interband transitions are usually observed, can substantially affect the value of the phase shift ψ(ω_0_). Nevertheless, according to Filatova *et al.* (1999 ▸, 2010 ▸), one can choose the integration domain to be large enough so as not to affect the physical results.

In the high-energy region (ω > ω_2_), where the photon energy exceeds the highest ionization potential of the mater­ial, the power law can be used,
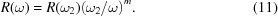
The main problem is the choice of the value of index *m*. It is well known that, at the frequencies exceeding the ionization potential of the *K*-edge, μ ≃ ω^−3^ is valid. For the reflection coefficient one can deduce that *R* ≃ *ω* ^−4^ under the same conditions. This law defines the greatest value of the index *m*. By inserting (11) into (8) one can obtain the addition to the phase shift from the high-energy region,
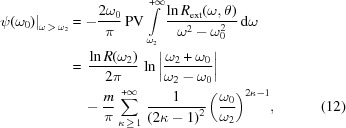
where ω_0_ is the frequency for which the phase shift is calculated; ω_2_ is the upper limit of the frequency range where the reflection coefficient is known; *R*(ω_2_) is the reflection coefficient at frequency ω_2_; *R*_ext_ is the extrapolation of the reflection spectrum outside of the experimental range to the region of high frequencies. As follows from the (12), the value of the addition depends linearly on the index *m* for each fixed frequency ω_0_ and is defined by the ratio ω_0_/ω_2_, which changes noticeably only over the wide frequency range and is nearly constant within the narrow range. We can conclude that the frequency positions of extrema in the reflection spectra do not depend on the value of the index *m*. Let us consider the spectral dependencies of the coefficients *a* and *b*, defined by the relations (7) on the index *m*. Since the phase shift ψ(*ω*_0_) depends linearly from the index *m* one can write 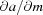 ≃ 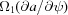 and 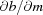 ≃ 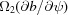, where Ω_1_ and Ω_2_ are proportionality factors. Then the derivative for coefficients *a* and *b* can be expressed as
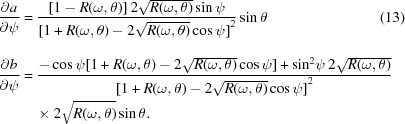
Since the phase shift can vary in the range 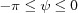 and the reflection coefficient is variable from unity to zero, it is obvious that 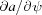 is always positive, which means that, at fixed reflection coefficient to smaller values of the phase shift, the lower values of the coefficient *a* correspond. One can conclude that the spectral dependencies of the coefficient *a* at different indexes *m* do not intersect and have the same behavior (shape). A completely different type of situation occurs for 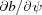. The values at which the derivative 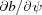 is zero satisfy the equation
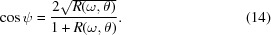
This means that the derivative in this case changes sign and the spectral dependencies of the coefficient *b*, obtained for different index *m*, are intersected leading to the redistribution of the intensity of the main peaks in the vicinity of the absorption edge depending on the value of index *m*. One can conclude that the choice of the extrapolation of the experimental reflection spectrum to the high energy range is a prime consideration in respect of determination of the optical constants of materials.

Filatova *et al.* (1999 ▸) found that, in the region of total external reflection (small grazing incidence angles) for extrapolation of the experimental reflection spectrum of SiO_2_ toward the high-energy region, where the photon energy exceeds the ionization potential of the Si *K*-edge, the relation *R*(ω) ≃ ω^−4^ can be used for energies defined by the relation 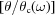 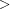 2 [θ_c_(ω) is a critical angle of total external reflection]. In the case of reflection spectra of heavy elements the relation 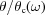 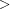 3.7 is more preferable (Filatova *et al.*, 2010 ▸).

For extrapolation of the experimental reflection spectrum toward the low-energy region (ω < ω_1_), different extrapolations were used, in particular according to linear and parabolic laws and along a straight horizontal line. It was established by Filatova *et al.* (1985 ▸) that the calculated data only slightly depend on the type of extrapolation in this region. The type of extrapolation in this region should be chosen from considerations of the smoothness of the cross-linking of the experimental spectrum with the extrapolating curve. In order to obtain a smooth cross-linking of the experimental spectra with the extrapolation curve, it was proposed to use (Filatova, Stepanov & Lukyanov, 1996 ▸; Filatova, Lukyanov, Blessing & Friedrich, 1996 ▸), in the low-energy range (ω < ω_1_), the following extrapolation,
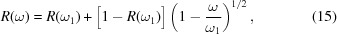
from the point 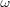 < 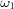 to *R* = 1 at 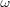 = 0.

## 4. Results and discussion   

**Figure 1:**
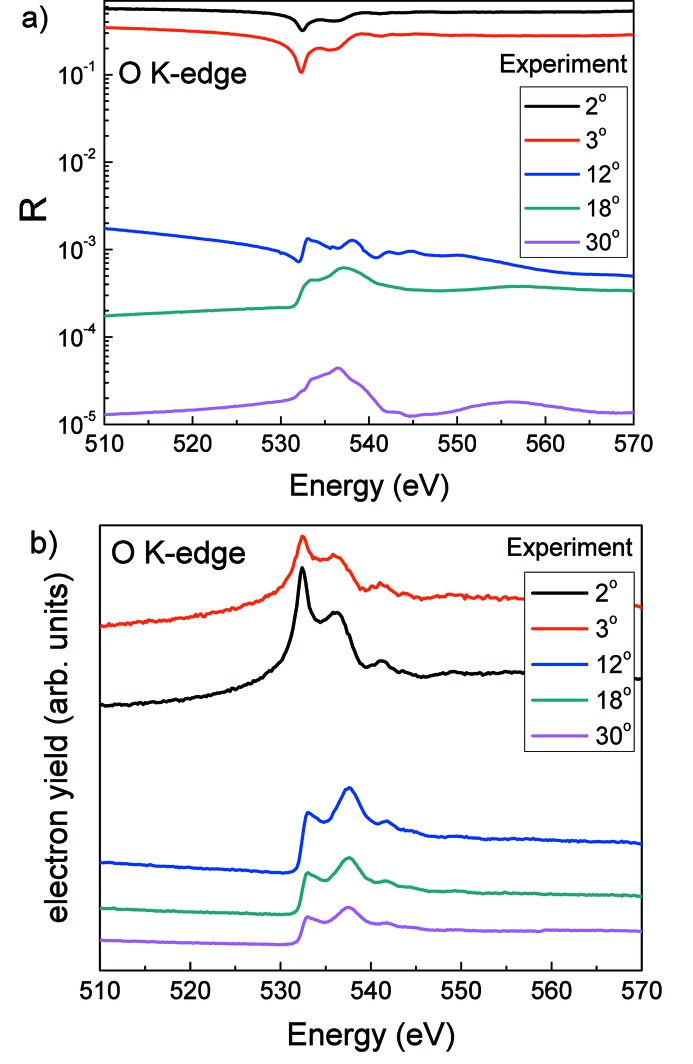
Reflection spectra (*a*) and TEY spectra (*b*) measured in the vicinity of the oxygen edge at different glancing angles for 5 nm-thick HfO_2_ film.

Fig. 1 ▸ shows the reflection spectra (RS) (panel *a*) and TEY spectra (panel *b*) of a 5 nm-thick HfO_2_ film measured at different glancing angles θ in the vicinity of the O *K*-absorption edge. Registration of the reflected beam and the drain current from the sample were carried out simultaneously in the case of each incident angle. Note that a completely different tendency in the evolution of the shape of the RS and TEY spectra with angle is traced. The TEY spectra measured at large angles reveal almost the same shape, which correlates well with the absorption spectra for HfO_2_ presented elsewhere (Lucovsky *et al.*, 2009 ▸; Kim *et al.*, 2011 ▸), whereas the spectra measured at small angles (2° and 3°) are different. Such a difference is caused by the high value of reflectivity at small glancing angles, which should undoubtedly have an effect on the TEY value according to formula (3).

Consideration of the reflection spectra reveals that only the spectra measured at 2° and 3° do not differ in shape. Rough estimates of the depth of formation of the reflected beam discussed by Filatova *et al.* (1998 ▸) point to the fact that at these glancing angles the HfO_2_ layer is thick enough for radiation to almost not penetrate through and so cannot reach the substrate. An estimation of the critical angle of the total external reflection gives a value of ∼4°. The shapes of the other spectra change catastrophically with increasing grazing incidence angle that is governed by interference of the waves reflected from the surface of the HfO_2_ layer and Si substrate.

Let us turn firstly to a detailed analysis of the measured reflection spectra and absorption spectra, calculated from experimental reflection spectra. Since the shape of the reflection spectra measured at large angles is distorted by the interference effect, we have used only the spectra measured at small incident angles to calculate the optical constants of the HfO_2_ layer. Note that the spectra were measured in a rather narrow energy region. To deduce the spectral dependence of the optical constants, we used the Kramers–Kronig relation and process explained above.

At the first step the reflection spectrum measured at the glancing angle of 2° was used. We used the fact that, in the range of normal dispersion (far from the absorption edges), the atoms in condensed systems can be considered as independent scattering dipoles; then the total atomic dipole moment is proportional to the average atomic factor of the medium, and the reflectivity in the energy region from 570 eV to high energies can be calculated on the basis of the atomic scattering factors from the tables of Henke *et al.* (1993 ▸). It should be clarified that in the work of Filatova *et al.* (2010 ▸) the optical constants of HfO_2_ film were calculated from reflection spectra measured at a glancing angle of 2° in the spectral region from 143 eV to 927 eV. It was established that at small glancing angles the relation *R*(*E*) ≃ *E* ^−4^ can be used for the extrapolation of the experimental reflection spectrum of HfO_2_ toward high energies, such that θ/θ_c_ > 3.7 [where θ_c_(ω) is a critical angle of total external reflection at *E*_ext_], that means that some absorption edges can be excluded from consideration.

The experimental spectrum from 570 eV to 3220 eV using the atomic scattering factors from the tables of Henke *et al.* (1993 ▸) was extended. In the low-energy range in order to obtain a smooth connection of the experimental spectra with the extrapolated curve we used the linear law to the point *R* = 1 at *E* = 0.

**Figure 2:**
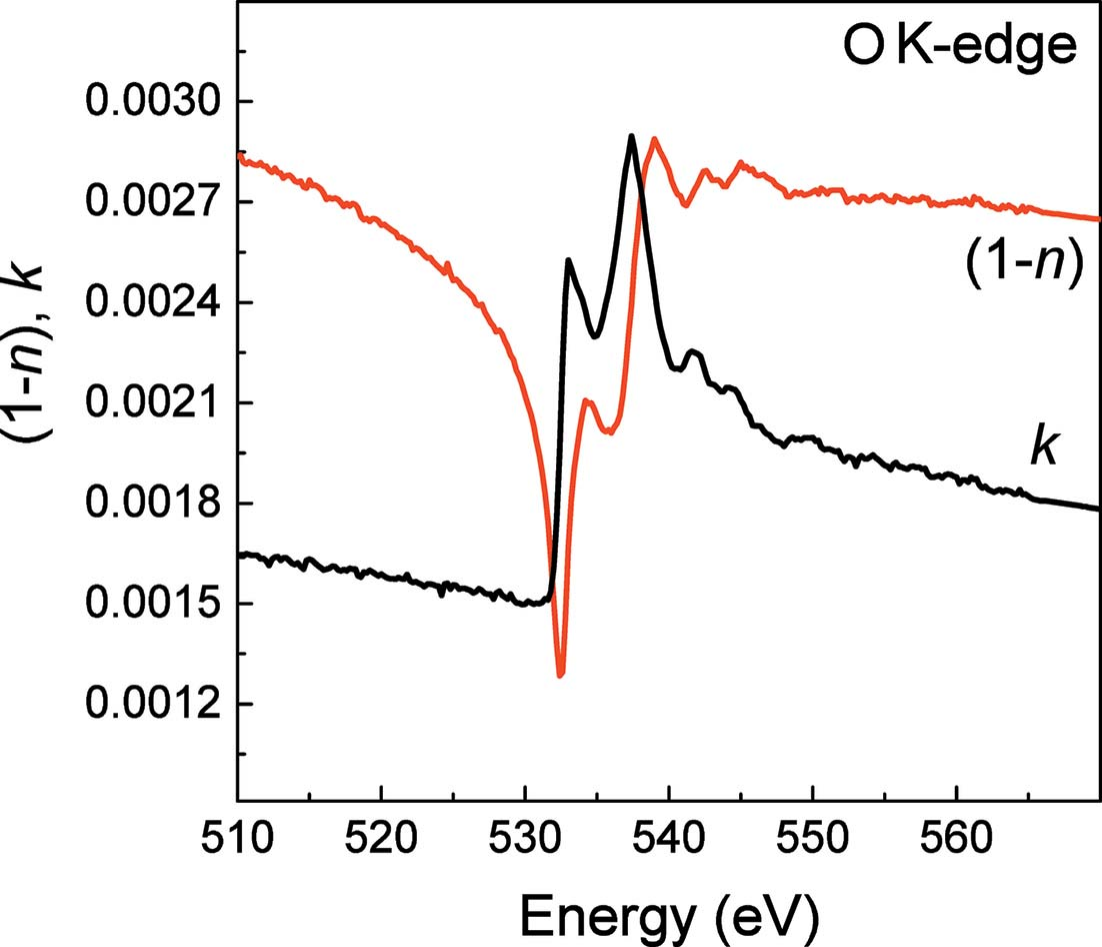
Spectral dependencies of the optical constants for HfO_2_ in the vicinity of the O *K*-absorption edge derived from the reflection spectrum (Fig. 1*a* ▸) measured at a glancing angle of θ = 2°.

Following Lucarini *et al.* (2005 ▸) and Filatova *et al.* (1999 ▸), the attenuation of the specular reflection coefficient due to roughness was taken into account by the Debye–Waller factor as follows,
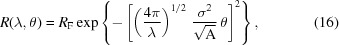
where *R*(ω,θ) is the reflectivity of a rough surface, *R*_F_ is the reflectivity of a perfect surface, σ is the r.m.s. roughness and *A* is the radius of correlation. The derived spectral dependencies of the optical constants *n* and *k* in the vicinity of the O *K*-absorption edge are shown in Fig. 2 ▸. A cross-check of the derived optical constants with the calculated ones on the basis of reflection spectra measured within a wide energy range and presented by Filatova *et al.* (2010 ▸) shows good agreement. Besides, a comparison of the derived optical constants with optical constants calculated from atomic scattering factors at energies far from the O *K*-absorption edge, where it is possible to use atomic scattering factor data from Henke *et al.* (1993 ▸), reveals a good correlation in their absolute values.

**Figure 3:**
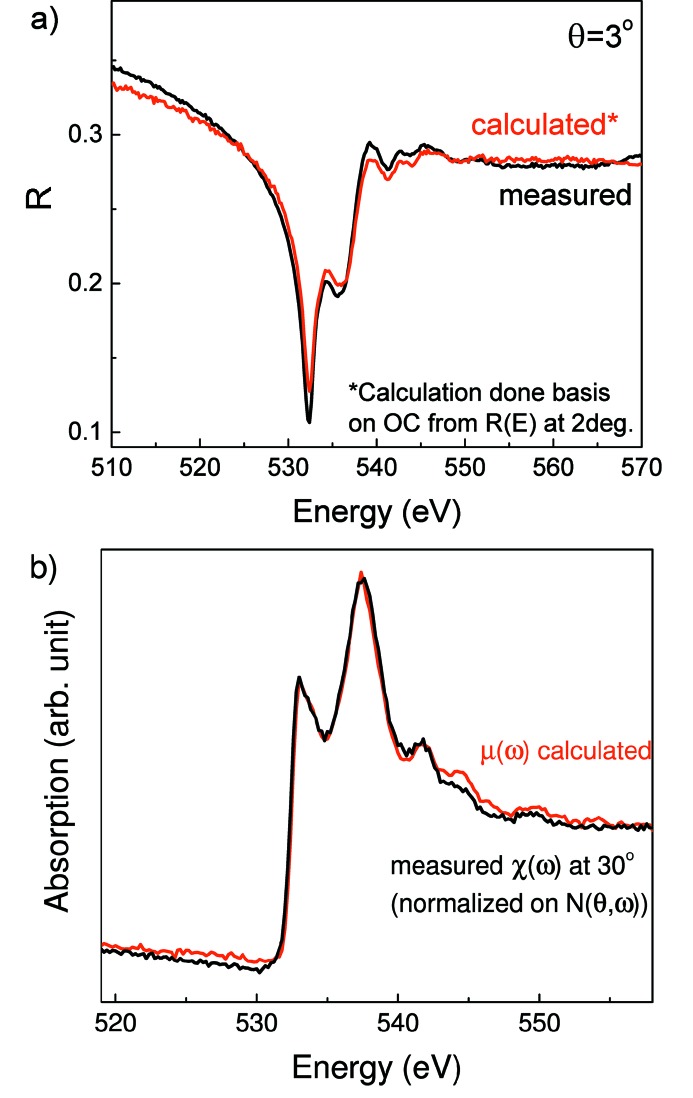
Comparison of the O *K*-reflection spectra measured at a glancing angle of 3° and calculated using the derived optical constants (*a*). Comparison of the O *K*-absorption spectra measured using the TEY method at θ = 30° and derived from calculated optical constants (*b*).

As was mentioned above, the implementation of the extrapolation of the reflection spectrum outside the experimental range is the most crucial issue in the process of calculating the optical constants. One of the purposes of the current work is to accurately study this problem. To this end, at the second step, we have calculated the O *K*-reflection spectrum at a glancing angle of 3° for a HfO_2_ layer using the derived optical constants and compared it with the reflection spectrum measured at θ = 3°. The calculated and measured O *K*-reflection spectra are shown in Fig. 3(*a*) ▸. The difference in absolute values of the measured and calculated reflection coefficients does not exceed 5%.

The absorption spectrum was calculated from the spectral dependence of the optical constant *k*(ω) using the equation 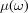 = 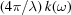. In Fig. 3(*b*) ▸ the calculated absorption spectrum μ(ω) is compared with the spectrum of the TEY measured at a glancing angle of 30° and normalized to a factor *N*(θ,ω). The meaning of the factor *N*(θ,ω) can be understood from equation (3). If, assuming that in the range of large glancing angles θ the relation 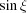 ≃ 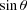 is valid, one can rewrite equation (3) in the form
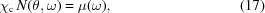
where
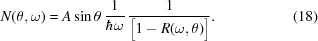
Parameter *A* is a constant and allows one to match the TEY spectrum with the calculated absorption spectrum. Obviously the constant *A* in *N*(θ,ω) includes both the normalization on parameters ∊ and *L* as follows from equation (3) and the characteristics of the experimental setup. In the ideal case, the presented spectra μ(ω) and normalized χ(ω) should coincide. As follows from Fig. 3(*b*) ▸, the spectra correlate well and the deviation in their absolute values does not exceed 3%. Taking into account that the presented spectra μ(ω) and χ(ω) were obtained independently, one can conclude that the technique developed by us for the calculation of optical constants from the reflection spectra allows a reasonable value of the optical constants to be derived even in the case of a narrow spectral range where the reflectivity was measured.

**Figure 4:**
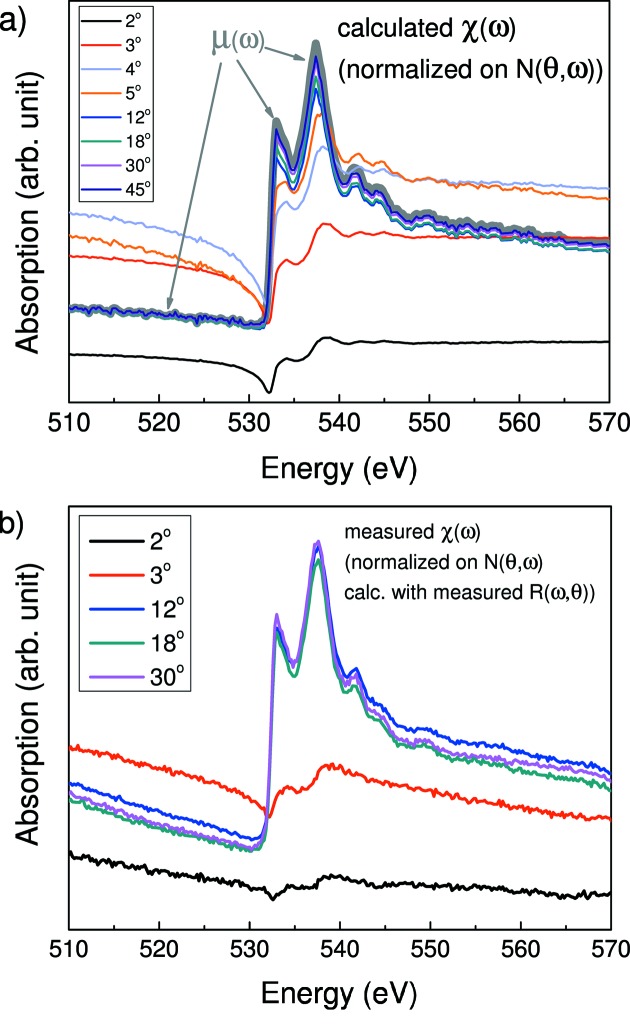
(*a*) TEY spectra of the 5 nm-thick HfO_2_ film for different glancing angles calculated within the simple model and normalized to *N*(θ,ω) using derived optical constants. (*b*) The absorption spectrum μ(ω) derived from the optical constant *k*(ω) is shown by a bold line. The measured TEY spectra (Fig. 1*b* ▸) normalized to *N*(θ,ω) calculated using measured *R*(θ,ω) (Fig. 1*a* ▸).

Fig. 4(*a*) ▸ shows the TEY spectra calculated for different glancing angles within the simple model using derived optical constants and normalized to factor *N*(θ,ω) with fixed parameter *A* and calculated *R*(θ,ω). On the same graph the absorption spectrum μ(ω) derived from the spectral dependence of the optical constant *k*(ω) is shown. Fig. 4(*b*) ▸ presents the measured TEY spectra from Fig. 1(*b*) ▸ which were normalized to factor *N*(θ,ω) with *R*(θ,ω) taken from Fig. 1(*a*) ▸ for each θ. As follows from comparison of Figs. 4(*a*) and 4(*b*) ▸, a good correlation between the spectra for large glancing angles is traced.

**Figure 5:**
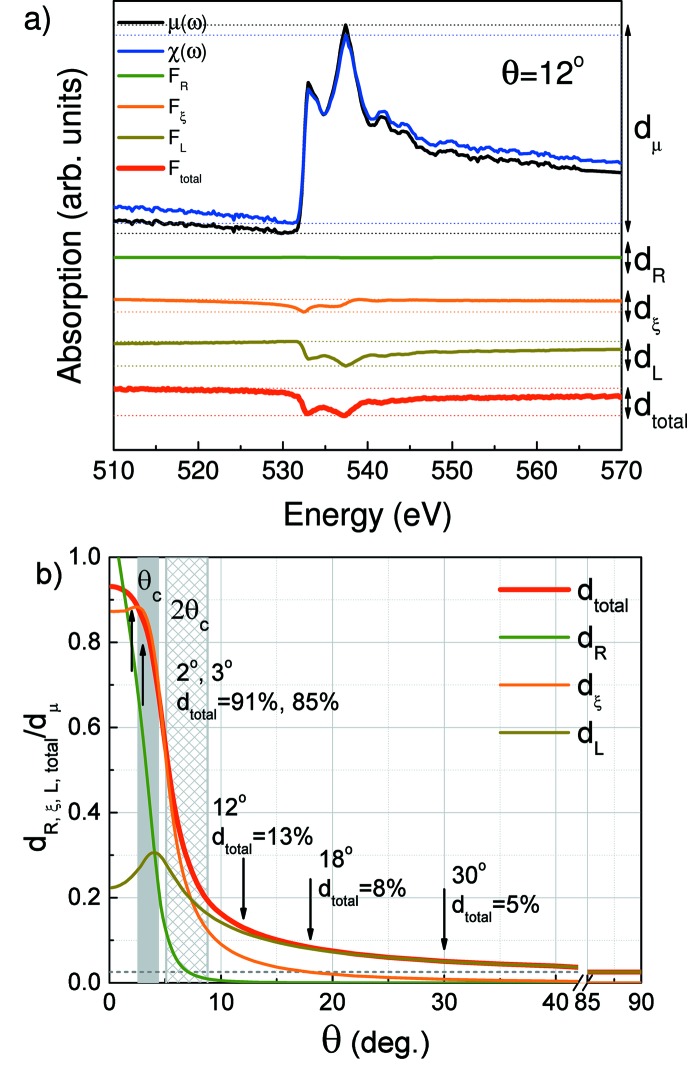
Panel (*a*) shows the spectral dependencies of the linear absorption coefficient μ(*E*), TEY [χ(*E*)] and factors *F*_R_, *F_ξ_*, *F*_L_ and *F*_total_ calculated for a glancing angle of 12° using derived optical constants (Fig. 2 ▸). Panel (*b*) shows the angular dependencies of parameters *d*_R_, *d*_ξ_, *d*_L_ and *d*_total_ estimated for factors *F*_R_, *F*_ξ_, *F*_L_ and *F*_total_, respectively. The grey area shows the range of critical angles θ_c_(*E*) in the photon energy region studied.

The careful comparison of the μ(ω) and *k*(ω) spectra for each θ angle used reveals a distinction, which we can introduce like a function: 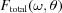 = 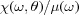, which decreases with increasing angle, but persists even at the largest angle used. To understand the reason for the observed tendency let us turn to the analysis of the expression (2) and represent it through several factors which in different ways depend on the angle and give different contributions to *F*_total_,
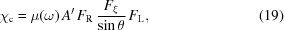
where
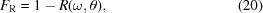


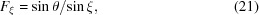


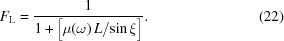


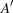
 is a constant, which is independent of photon energy and glancing angle and is defined by the experimental setup. Fig. 4(*a*) ▸ shows the spectral dependencies of the linear absorption coefficient μ(*E*), TEY [χ(*E*)] and factors *F*_R_, *F*_ξ_, *F*_L_ and *F*_total_ calculated for a glancing angle of 12° using derived optical constants (Fig. 2 ▸). To obtain a numerical estimation of the introduced factors *F*_R_, *F*_ξ_, *F*_L_ in *F*_total_ it is convenient to introduce the parameters *d*_R,ξ,L,total_, reflecting the difference between maximum and minimum in spectral dependence of each factor *F*_R,ξ,L,total_, and *d*_μ_ for μ(ω) (Fig. 5*a* ▸).

The angular dependencies for parameter *d*_R,ξ,L,total_ were calculated using the derived optical constants. These dependencies were normalized to *d*_μ_ and are plotted in Fig. 5(*b*) ▸. Analysis of the calculated dependencies allows to trace the contribution from each factor in *F*_total_ (through analysis of the *d*_total_ parameter) at different glancing angles. As can be seen in the region of total external reflection (at glancing angles smaller than the critical angle θ_c_), the value of *d*_total_ is close to 90–80% and is formed mainly due to *d*_R_ and *d*_ξ_. One can conclude that in this angular region the fine structure of the TEY spectrum is distorted not only by the effect of X-ray reflection but at the same extent by the effect of X-ray refraction. This is a key issue because the reflectivity can be easily measured and taken into account while the direct measurement of refraction is impossible for thick samples in the soft X-ray region.

Analysis of *d*_total_ in the angular region θ_c_< θ < 2θ_c_ reveals the change of its value from 80% to 20%, which is predominantly formed by *d*_ξ_ (that means by the effect of refraction). For angles θ > 2θ_c_, *d*_total_ decreases to 3% and is defined mostly by *d*_L_. Let us look at formula (22). Taking into account that at large glancing angles sin(ξ) weakly depends on the energy and 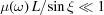, the factor *F*_L_ can be presented as
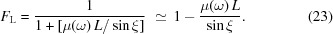
One can see from relation (23) that at large glancing angles (for angles θ > 2θ_c_) the spectral dependence of factor *F*_L_ is inverse to the μ(*E*) spectrum, which points to the coincidence of energy positions of features in the μ(*E*) and χ(*E*) spectra but differences in their amplitudes to within a few percent. The amplitude of the χ(*E*) spectrum is always lower than that of μ(*E*). Analysis of the spectral dependencies of μ(*E*), χ(*E*) and factors *F*_R_, *F*_ξ_, *F*_L_ and *F*_total_ calculated for a glancing angle of 12° using derived optical constants (Fig. 5*a* ▸) confirms this.

## 5. Conclusions   

The technique developed by us for the calculation of optical constants from the reflection spectra allows a reasonable value for optical constants to be derived even in the case of a narrow spectral range where the reflectivity was measured. The analysis carried out has been corroborated by the fact that in the soft X-ray region at sufficiently large glancing angles we can neglect reflection and refraction and express the absorption coefficient μ(θ,ω) in terms of the regular absorption coefficient μ(ω). Also, it is absolutely warranted to use the data obtained by TEY in this region. At the same time it was found that the effect of refraction on the TEY spectrum is greater than that of reflection and extends into the angular region up to angles 2θ_c_. Within angles less than the critical angle both the reflection and refraction distort the shape of the TEY spectrum.
